# Identification of genomic regions for anthracnose resistance in the Andean mapping population of common bean derived from a cross of genotypes Lusaka and Mwezi Moja

**DOI:** 10.3389/fpls.2026.1897486

**Published:** 2026-10-05

**Authors:** Mukuni Nkandela, Swivia Hamabwe, Gideon Munduwe, Alvaro Soler-Garzón, Karen Cichy, Travis Parker, Kelvin Kamfwa

**Affiliations:** 1 Dept. of Plant Sciences, University of Zambia, Lusaka, Zambia; 2 Grain Legume Genetics and Physiology Research Unit, United States Department of Agriculture–Agricultural Research Service, Prosser, WA, United States; 3 USDA-ARS, Sugarbeet and Bean Research Unit, East Lansing, MI, United States; 4 Dept. of Plant Sciences, University of California, Davis, Davis, CA, United States

**Keywords:** candidate genes, *Colletotrichum lindemuthianum*, pathogen race, quantitative trait locus, recombinant inbred lines

## Abstract

Anthracnose (ANTH), caused by *Colletotrichum lindemuthianum*, is a major disease of common bean (*Phaseolus vulgaris* L.) and an important constraint to bean production in Zambia and other countries. The high genetic variability of the pathogen and the occurrence of numerous physiological races complicate the development of varieties with durable resistance. The objectives of this study were to (i) characterize isolates of *C. lindemuthianum* collected from Zambia into physiological races and (ii) identify genomic regions associated with resistance to anthracnose in a recombinant inbred line (RIL) population. Four isolates collected from two major bean-producing districts of Zambia were characterized as races 7, 15, 17, and 291 using standard differential cultivars. A population of 110 F_5:6_ RILs derived from a cross between Mwezi Moja, a Kenyan variety with purple speckled seed color, and Lusaka, a Zambian landrace with manteca yellow seed color, was evaluated for resistance to the four races and one uncharacterized isolate A23-13. RIL LM145 exhibited high resistance to all four races and the uncharacterized isolate A23-13. Genotyping of the population with the BARCBean12K SNP array followed by composite interval mapping identified four QTL: ANT1.1^LM^, ANT2.1^LM^, ANT4.1^LM^, and ANT10.1^LM^. ANT1.1^LM^ explained 60.5% and 57.7% of the phenotypic for resistance to race 17 and A23-13, respectively; ANT2.1^LM^ explained 4.2% for A23-13; ANT4.1^LM^ explained 75.5% for race 291; and ANT10.1^LM^ explained 18.9% and 25.9% for races 7 and 15, respectively. ANT1.1^LM^ and ANT4.1^LM^ co-localized with the known resistance loci *Co-1* and *Co-3*, respectively. Pyramiding of major resistance loci identified in this study may contribute to broader resistance to diverse races of *C. lindemuthianum*.

## Introduction

Anthracnose (ANTH), caused by the hemibiotrophic fungus *Colletotrichum lindemuthianum*, is among the most destructive diseases of common bean (*Phaseolus vulgaris* L.), a crop that plays an important role in sustainable agricultural systems and food security in many countries (Uebersax et al., 2022). The pathogen is primarily seedborne, with dissemination occurring largely through the planting of infected seed (Kelly and Vallejo, 2004). Under favorable environmental conditions, which is typically high humidity and moderate to cool temperatures, ANTH can cause yield losses of up to 100% in susceptible cultivars. The disease affects all aerial plant parts, producing characteristic lesions on leaves, stems, and pods. In Zambia, ANTH is a major production constraint, with a recent study reporting particularly high incidence and severity in northern regions where bean production is concentrated (Mulube et al., 2026a). ANTH management is challenging particularly in Africa because most smallholder farmers rely on farm-saved seed (Mulube et al., 2026a), which facilitates pathogen carryover between seasons. Although fungicidal control can be effective, it is often unaffordable and raises health and environmental concerns. Consequently, the deployment of host plant resistance remains the most practical and sustainable management strategy for ANTH.

A major challenge in breeding for ANTH resistance is the extensive genetic variability of *C. lindemuthianum*. More than 300 races have been reported worldwide (Nunes et al., 2021), and over 80 races have been documented in Zambia alone (Zulu, 2005; Kachapulula, 2010; Sansala et al., 2024; Kachapulula et al., 2025; Mulube et al., 2026b). Multiple races may occur within a single farmer’s field, underscoring the pathogen’s evolutionary potential within a small geographic area (Sansala et al., 2024). *C. lindemuthianum* is classified into Andean and Middle American gene pool because of its co-evolution of its infection pattern on the two gene pools of common bean (Balardin and Kelly, 1998); da Silva Dambroz et al., 2023). Andean races are typically more virulent on Andean genotypes, whereas Middle American races tend to have more broad virulence infecting both Andean and Middle American though more virulent on Middle American germplasm (Paulino et al., 2022). Mixed races capable of infecting both gene pools also occur. In countries such as Zambia, where Andean beans predominate, Andean and mixed races are more prevalent (Sansala et al., 2024). The extensive genetic diversity of *C. lindemuthianum* poses a major challenge to resistance breeding because resistance is largely race-specific, and follows a gene-for-gene interaction, meaning that no single resistance gene confers universal protection.

Major ANTH resistance loci (designated *Co* loci) and quantitative trait loci (QTL) have been identified across all eleven chromosomes of the bean genome. These *Co* loci typically encode nucleotide-binding leucine-rich repeat (NB-LRR) proteins that occur in gene clusters and confer race-specific (vertical) resistance (Mungalu et al., 2020; Mulube et al., 2026b; Sinkala et al., 2024; Kachapulula et al., 2025; Kuwabo et al., 2023);. The *Co* loci are broadly associated with either Andean or Middle American gene pools, reflecting host–pathogen co-evolution. In addition to these major genes, both major- and minor-effect quantitative trait loci (QTL) contributing to partial (horizontal) resistance have been reported (Mulube et al., 2026b; Sinkala et al., 2024; Kachapulula et al., 2025; Kuwabo et al., 2023; Mungalu et al., 2020; Banoo et al., 2020). The combination of major resistance genes with minor-effect QTL is considered essential for achieving durable resistance. Genome-wide association studies conducted in diverse panels, including the Andean Diversity Panel (Mulube et al., 2026b; Zuiderveen et al., 2016), Middle American Panel (Sinkala et al., 2024), and Yellow Bean Collection (Kuwabo et al., 2023), as well as linkage mapping studies in biparental populations, have identified novel genomic regions associated with resistance to specific pathogen races.

Common bean market classes are primarily defined by seed coat color and associated consumer preferences, which strongly influence adoption, marketability, and breeding priorities. In Eastern and Southern Africa, the yellow bean market class has gained prominence because of its relatively fast cooking time, palatable texture, and favorable nutritional profile, making it highly preferred by both rural and urban consumers (Sichilima et al., 2016; Wiesinger et al., 2018). The Kabulangeti type, characterized by a distinctive purple speckled seed coat, is likewise an important market class in Zambia and Tanzania due to its culinary quality and established demand in local markets (Hamabwe et al., 2024). Improving ANTH resistance in these preferred classes is therefore essential to sustain productivity while maintaining traits valued by consumers.

A biparental population of 133 F_5_ recombinant inbred lines (RILs), derived from a cross between the yellow-seeded genotype Lusaka and the Kabulangeti variety Mwezi Moja. However, this variation has not been investigated under controlled exposure to characterized races of *C. lindemuthianum*. Without race-specific evaluation, it is not possible to determine whether the observed resistance is broad, race-specific, or quantitatively inherited. Moreover, the genetic basis underlying this resistance has not yet been investigated, limiting the ability to efficiently utilize these materials in breeding.

QTL associated with resistance to specific races would enable the deployment of molecular markers to guide selection, thereby reducing reliance on laborious phenotypic screening and improving breeding efficiency. Such information is particularly valuable for introgressing resistance into market-preferred yellow and Kabulangeti bean types. The objectives of this study were: (*i*) characterize four isolates of *C. lindemuthianum* into races, and (*ii*) map the genomic regions for resistance to ANTH in the Lusaka × Mwezi Moja RIL population.

## Materials and methods

### Plant materials

For race characterization of the pathogen isolate, a set of 12 ANTH race differential cultivars described by Pastor-Corrales (1991) was used (**Table 1**). These cultivars are universally used for the characterization of *C. lindemuthianum* races. The differentials originate from both the Andean and Middle American gene pools and carry a range of ANTH resistance genes. Each differential cultivar is assigned a binary value, and the cumulative binary value of susceptible differentials to a particular isolate determines the race number of that isolate.

**Table 1:** Reaction of the twelve differential cultivars to inoculation with *Colletotrichum lindemuthianum* races 7, 15, 17, and 291 used to determine pathogen race identity.

Race differential	Gene pool	Gene	Binary number	Race/isolate name			
				7	15	17	291
Michelite	MA	*Co-11*	1	9	9	9	9
MDRK	A	*Co-1*	2	9	9	1	9
Perry Marrow	A	*Co-1^3^*	4	9	9	1	1
Cornell 49242	MA	*Co-2*	8	1	9	1	1
Widusa	A	*Co-1^5^*	16	1	1	9	1
Kaboon	A	*Co-1^2^*	32	1	1	1	9
Mexico 222	MA	*Co-3*	64	1	1	1	1
PI 20262	MA	*Co-3^3^; Co-4^3^*	128	1	1	1	1
TO	MA	*Co-4*	256	1	1	1	9
TU	MA	*Co-5*	512	1	1	1	1
AB 136	MA	*Co-6; Co-8*	1024	1	1	1	1
G2333	MA	*Co-4^2^, Co-5^2^*	2048	1	1	1	1

MA, Middle American gene pool; A, Andean gene pool.

A total of 133 F5:6 RILs derived from the cross ‘Lusaka’ × ‘Mwezi Moja’ (LM) were genotyped. Of these, 110 RILs were phenotyped (**Supplementary Table 1**) and used for QTL mapping, while 23 RILs were excluded due to insufficient seed availability for evaluation. The RILs were developed using the single seed descent method. Lusaka is a popular Andean landrace from Zambia with a Type I growth habit and a Manteca yellow seed color. Mwezi Moja is an Andean variety from Kenya with a purple-speckled seed color and a Type I growth habit, and it is identified as ADP466 within the Andean Diversity Panel (Cichy et al., 2015). The two parental lines exhibit differential responses to infection by *C. lindemuthianum* races.

The differential cultivar G2333, previously reported to be highly resistant to many ANTH races including those occurring in Zambia, was used as a resistant check. The landrace Kabulangeti, which has widely been reported to be susceptible to ANTH in Zambia, was used as a susceptible check (Mungalu et al., 2020).

### Isolate collection and characterization

Common bean pods showing symptoms of ANTH infection were collected from bean fields at the Zambia Agricultural Research Institute (ZARI) research stations in Mpika (Muchinga Province) and Kasama (Northern Province). These stations are widely used as ANTH hotspot screening sites by several common bean breeding programs in Zambia and are located in major bean-producing districts.

The infected pods were transported to the University of Zambia Plant Pathology Laboratory, where fungal isolations were conducted following the protocol described by Sansala et al. (2024). Small sections of infected tissue (0.5–2 mm) were excised from lesions and surface sterilized using 1% sodium hypochlorite and 70% ethanol for two minutes. The tissue pieces were then rinsed in distilled water and placed on paper towels for approximately 45 minutes to air dry. The dried tissues were transferred onto potato dextrose agar (PDA) medium (39 g L^−^¹) in Petri dishes and incubated in darkness for 10 days to allow fungal growth and sporulation. Single-spore isolation was then conducted by transferring individual spores from sporulating cultures onto fresh PDA plates.

Five single-spore isolates were obtained and designated as A23-1, A23-2, A23-3, A23-4, and A23-13. These isolates were incubated in darkness for another 10 days to allow sporulation. To prepare inoculum suspensions, distilled water was added to sporulated cultures and the spores were gently scraped using a glass rod. The suspension was filtered through cheesecloth to remove mycelial fragments. Spore concentration was adjusted to 1.2 × 10^6^ spores mL^−^¹ using a hemocytometer. Tween-20 (10 μL per 100 mL) was added to the suspension to improve spore dispersion.

### Plant inoculation and disease evaluation

Seedlings were raised in Styrofoam trays containing 60 wells, each measuring 5 × 5 × 5 cm. Two seeds of each genotype were planted per well using a completely randomized design (CRD) with three replications. Each well containing two seedlings constituted an experimental unit, resulting in six seedlings per genotype across the three replications.

Seedlings were grown until primary leaves were fully expanded, after which inoculation was conducted using a handheld sprayer. Leaves were sprayed thoroughly on both the upper and lower surfaces until runoff occurred. After inoculation, seedlings were kept at room temperature for two hours to allow the inoculum to dry. The trays were then transferred to a high humidity chamber (95–100% relative humidity) maintained at 24–26 °C with a 12-hour photoperiod. Seedlings remained in the chamber for 72 hours and were subsequently moved to greenhouse benches maintained at 28–30 °C for five days to allow disease development.

ANTH severity was scored on each seedling using the 1–9 disease rating scale described by van Schoohoven and Pastor-Corrales (1987), where: 1 = no visible symptoms, 2–3 = limited necrotic lesions, 4–8 = small to large sporulating lesions, and 9 = severely infected or dead seedlings. Genotypes with scores of 1–3 were classified as highly resistant, 4–6 as moderately resistant, and 7–9 as susceptible. The disease scores were averaged per genotype and used in QTL mapping.

The race differential cultivars and the RIL population were planted, inoculated, and incubated simultaneously in the humidity chamber to enable concurrent isolate race characterization and phenotyping of the mapping population.

The isolate A23–13 was not race-characterized because it was collected later than the other isolates included in the study. At the time of its collection, seed of the 12 differential cultivars required for race determination was no longer available, preventing its inclusion in the race characterization analysis.

### Phenotypic data analysis

Anthracnose severity was recorded on the ordinal 1–9 scale. Because these scores are ordinal, inference from parametric analyses was interpreted cautiously. For each race and isolate, genotype effects were evaluated using the replicated disease severity observations. Log transformation was used in the original ANOVA to reduce departures from normality, and model residuals were examined for major departures from normality. The primary genetic analyses were based on genotype mean disease scores calculated from the six seedlings evaluated per genotype. Frequency distributions of the untransformed severity scores were used to describe phenotypic segregation within the RIL population. Broad-sense heritability (H²) was estimated from variance components on the raw disease-score scale using the lme4 package in R, with genotype treated as a random effect in the following model:


$$H^{2}=\frac{\sigma_{g}^{2}}{\sigma_{g}^{2}+\sigma_{e}^{2}/r}$$

Where *H^2^* is broad-sense heritability, $\sigma_{g}^{2}$ was variance component for genotype, and $\sigma_{e}^{2}$ was error variance component and *r* is the number of replications.

### Genotyping, linkage map construction, and QTL analysis

DNA extraction for the 133 RILs and their parental lines was conducted at Michigan State University using the NucleoSpinTM Plant II kit (Macherey-Nagel GmbH & Co., Dueren NRW, Germany). Plants were grown to the first trifoliate stage, and leaf tissue from a single plant per RIL was collected for DNA extraction. The extracted DNA samples were shipped to the USDA-ARS Beltsville Agricultural Research Center (Maryland, USA) for genotyping using the Illumina BARCBean12K BeadChip, which assays 11,292 SNP markers. SNP genotyping was conducted using the Illumina Infinium HD Assay Ultra protocol (Illumina Inc., San Diego, CA) and SNP calling (**Supplementary Table 2**) was performed using the Illumina GenomeStudio software. To ensure accurate mapping, SNP positions were updated through alignment against the Andean G19833 *v*2.1 *P. vulgaris* reference genome assembly (Schmutz et al., 2014).

A genetic linkage map was built for the LM RIL Population using MapDisto version 2.17 (Lorieux, 2012). After quality filtering, 731 markers were retained. Redundant SNP markers were removed, such as markers linked at 0 cM within a 100 kb physical distance, along with markers exceeding 10% missing data, heterozygosity greater than 0.1, or showing significant segregation distortion (*p* < 0.01). Linkage maps were generated using a maximum recombination fraction (rmax) of 0.24 and a minimum LOD score (LODmin) of 3, applying the Kosambi mapping function. SNP markers were ordered within linkage groups based on their physical positions according to chromosome assignments from the G19833 *v*2.1 *P. vulgaris* reference genome.

The resulting genetic map was comprised of 11 linkage groups corresponding to the common bean chromosomes (Pv01–Pv11). Linkage group sizes ranged from 88.5 cM (Pv10) to 242.1 cM (Pv09), with a total map length of 1783.8 cM and an average marker spacing of 2.4 cM. The number of SNPs per linkage group ranged from 29 (Pv10) to 93 (Pv02), with an average of 68 markers per chromosome.

QTL analyses for resistance to the five characterized races of *C. lindemuthianum* and one uncharacterized isolate were conducted using composite interval mapping (CIM) implemented in R/qtl in the R statistical environment (Broman et al., 2003). The analysis used the following parameters: (*i*) Kosambi mapping function, (*ii*) Haley-Knott regression and (*iii*) three SNP markers selected using the default R/qtl cofactor selection procedure were used as covariates. Significance thresholds for logarithm of odds (LOD) scores were determined using 1000 permutation tests for each race. The permutation-derived LOD thresholds for each race/isolate are provided in **Table 2**. QTL intervals were defined using Bayesian credible interval analysis. Linkage maps with identified QTL positions were visualized using the R package LinkageMapView.

**Table 2:** Quantitative trait loci (QTL) associated with resistance to *Colletotrichum lindemuthianum* races 7, 15, 17, and 291, and isolate A23–13 identified in a recombinant inbred line (RIL) population derived from a cross between Lusaka and Mwezi Moja.

QTL name	CHR	Race/ isolate	QTL peak (Mbp)	QTL interval (Mbp)	LOD	LOD threshold	*R^2^* (%)	Effect	Source
ANT1.1	Pv01	17	49.5	49.3-49.8	19.3	3.05	60.5	3.8	Mwezi Moja
ANT1.1	Pv01	A23-13	49.6	49.3-49.7	19.8	3.15	57.7	2.8	Mwezi Moja
ANT2.1	Pv02	A23-13	41.0	2.3-47.7	3.7	3.15	4.2	0.7	Lusaka
ANT4.1	Pv04	291	0.3	0.1-0.7	39.0	2.92	75.5	3.3	Mwezi Moja
ANT10.1	Pv10	7	41.5	39.6-41.6	4.5	3.08	18.9	6.6	Lusaka
ANT10.1	Pv10	15	41.0	39.6-41.3	5.2	3.16	25.9	6.4	Lusaka

QTL, Quantitative Trait Locus; Mbp, Million base pairs; LOD, logarithm of odds; LOD Threshold refers to the permutation-derived LOD threshold for each race; CHR=Chromosome; *R^2^* = proportion of phenotypic variance explained by the QTL; % = Percentage.

### Candidate gene identification

Candidate genes associated with detected QTL were identified using JBrowse to scan the *Phaseolus vulgaris* genome *v*2.1 available on Phytozome *v*14. Genes located within 250 Kbp upstream or downstream of the peak SNP marker were considered potential candidates.

Priority was given to genes previously reported or predicted to be involved in plant disease resistance pathways, following criteria similar to those described by Mungalu et al. (2020).

## Results

### Phenotypic evaluation of RILs for ANTH resistance

The four isolates A23-1, A23-2, A23-3, and A23–4 were characterized as races 7, 15, 17, and 291, respectively (**Table 1**). All four races were classified as mixed races, as they were virulent on both Andean differentials (MDRK, Perry Marrow, Kaboon, and Widusa) and Middle American differentials (Michelite and TO).

The resistant and susceptible checks were consistently resistant and susceptible, respectively, to all four races and the uncharacterized isolate throughout the experiment, confirming the reliability of the inoculation procedure.

Analysis of variance revealed significant (*P* < 0.05) variation among the RILs in their responses to inoculation with the four races and the isolate (**Table 3**). The severity scores for the 110 RILs for the four races and one isolate are shown in **Supplementary Table 1**. In addition, *t*-test comparisons showed that the two parental lines differed significantly in their reactions to all races and the isolate (**Table 3**).

**Table 3:** Mean and range of anthracnose severity scores for the parental lines Lusaka and Mwezi Moja, the checks G2333 and Kabulangeti, and the recombinant inbred line (RIL) population following inoculation with *Colletotrichum lindemuthianum* races (7, 15, 17, and 291) and uncharacterized isolate A23-13. .

Race	Parents			Checks		RILs			
	Lusaka	Mwezi	*t-*Test	G2333	Kab	Mean	Range	ANOVA	*H^2^ (%)*
Race 7	5	7.7	*	1	9	6.8	1.0-9.0	***	94.5
Race 15	3.3	8.5	**	1	9	6.8	1.0-9.0	***	95.5
Race 17	6	1	***	1	9	3.5	1.0-9.0	***	95.2
Race 291	9	2	***	1	9	5.7	1.0-9.0	***	96.6
A23-13	5.3	1	***	1	9	4.5	1.0-9.0	***	96.9

Broad-sense heritability (*H²*) estimates for anthracnose severity are also presented.

Severity scores were on a scale of 1 – 9; G2333 = resistant check; Kab (Kabulangeti), susceptible check; ANOVA, Analysis of Variance; * = 0.05 (level of significance); ** = 0.01 (level of significance) and *** = 0.001 (level of significance); n, number; RILs, Recombinant Inbred Lines; *H^2^*=Broad Sense Heritability.

For race 7, the mean ANTH severity score across the RIL population was 6.8, and the distribution of severity scores was skewed toward susceptibility (**Figure 1**). A similar pattern was observed for race 15, which also had a population ANTH severity score of 6.8 (**Figure 1**; **Table 3**). Lusaka exhibited a moderately resistant reaction to race 7, whereas Mwezi Moja was highly susceptible. In contrast, Lusaka was highly resistant to race 15, while Mwezi Moja remained highly susceptible.

**Figure 1:**
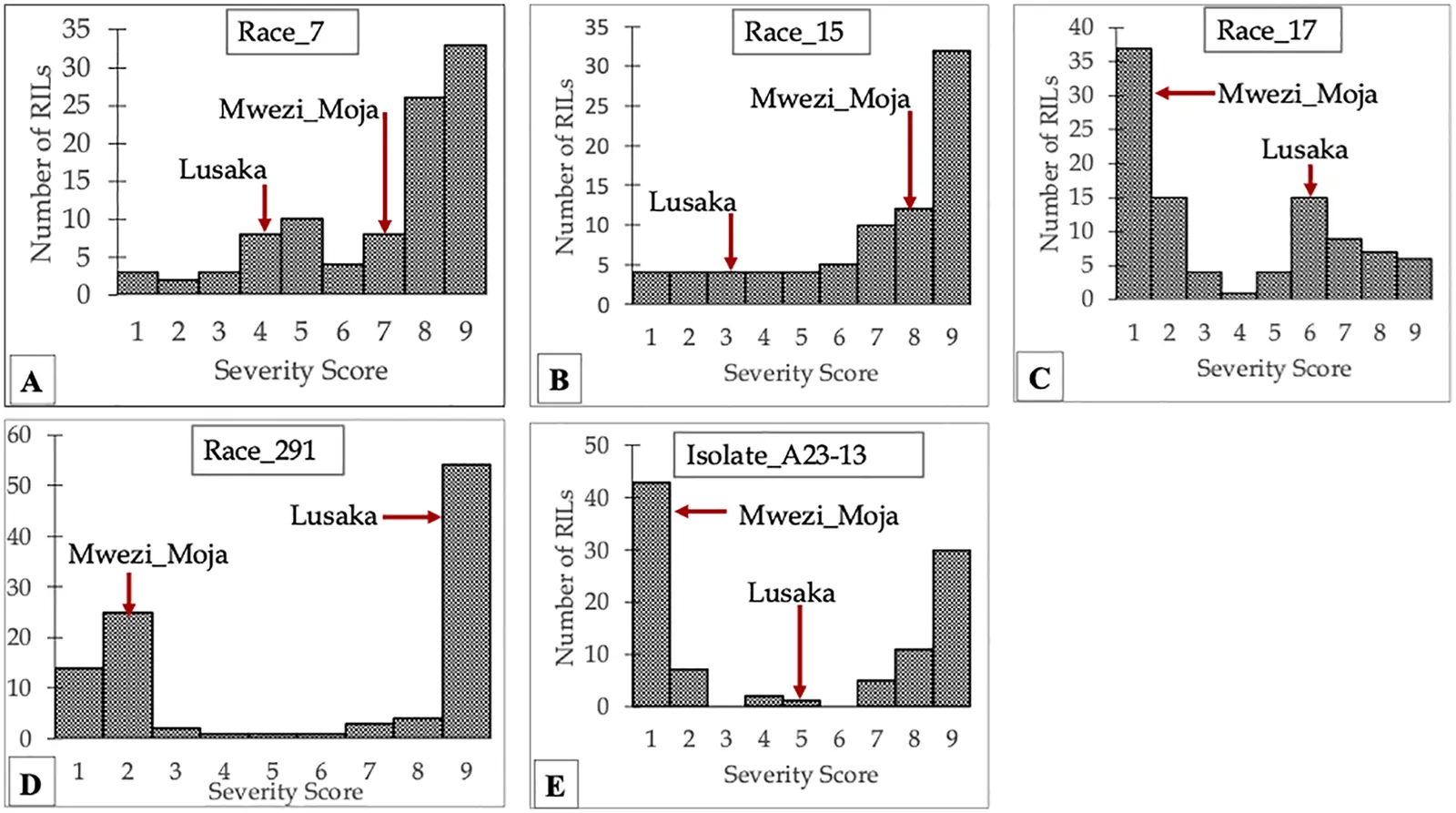
Frequency distributions of severity scores for *Colletotrichum lindemuthianum* race 7 **(A)**, 15 **(B)**, 17 **(C)**, 291 **(D)** and isolate A23-13 **(E)** inoculated on recombinant inbred lines derived from a cross of Lusaka and Mwezi Moja.

For race 17, the severity score distribution was skewed toward resistance (**Figure 1**), with a population mean of 3.5. Under this race, Mwezi Moja displayed a resistant reaction, while Lusaka was susceptible.

The phenotypic distributions for race 291 (**Figure 1**) and the uncharacterized isolate A23-13 (**Figure 1**) were bimodal, suggesting the involvement of major resistance loci. For both cases, Mwezi Moja exhibited high resistance, whereas Lusaka showed a susceptible reaction to race 291 but was moderately resistant to isolate A23-13.

Transgressive segregation for both resistance and susceptibility were observed among the RILs for all races and the isolate, indicating the presence of complementary resistance and susceptibility alleles in the parental lines. Among the RILs, LM145 was the only line that displayed superior resistance to all four races and the isolate.

Broad-sense heritability estimates for ANTH resistance were generally high, ranging from 94.5% for race 7 to 96.9% indicating that a large proportion of the phenotypic variation was attributable to genetic effects.

### QTL mapping and positional candidate gene identification

A total of four quantitative trait loci (QTL) associated with resistance to *Colletotrichum lindemuthianum* together with positional candidate genes were identified as the genetic basis of resistance to four races (7, 15, 17 and 291) and isolate A23–13 in the RIL population (**Table 2**; **Figure 2**; **Supplementary Table 2**).

**Figure 2:**
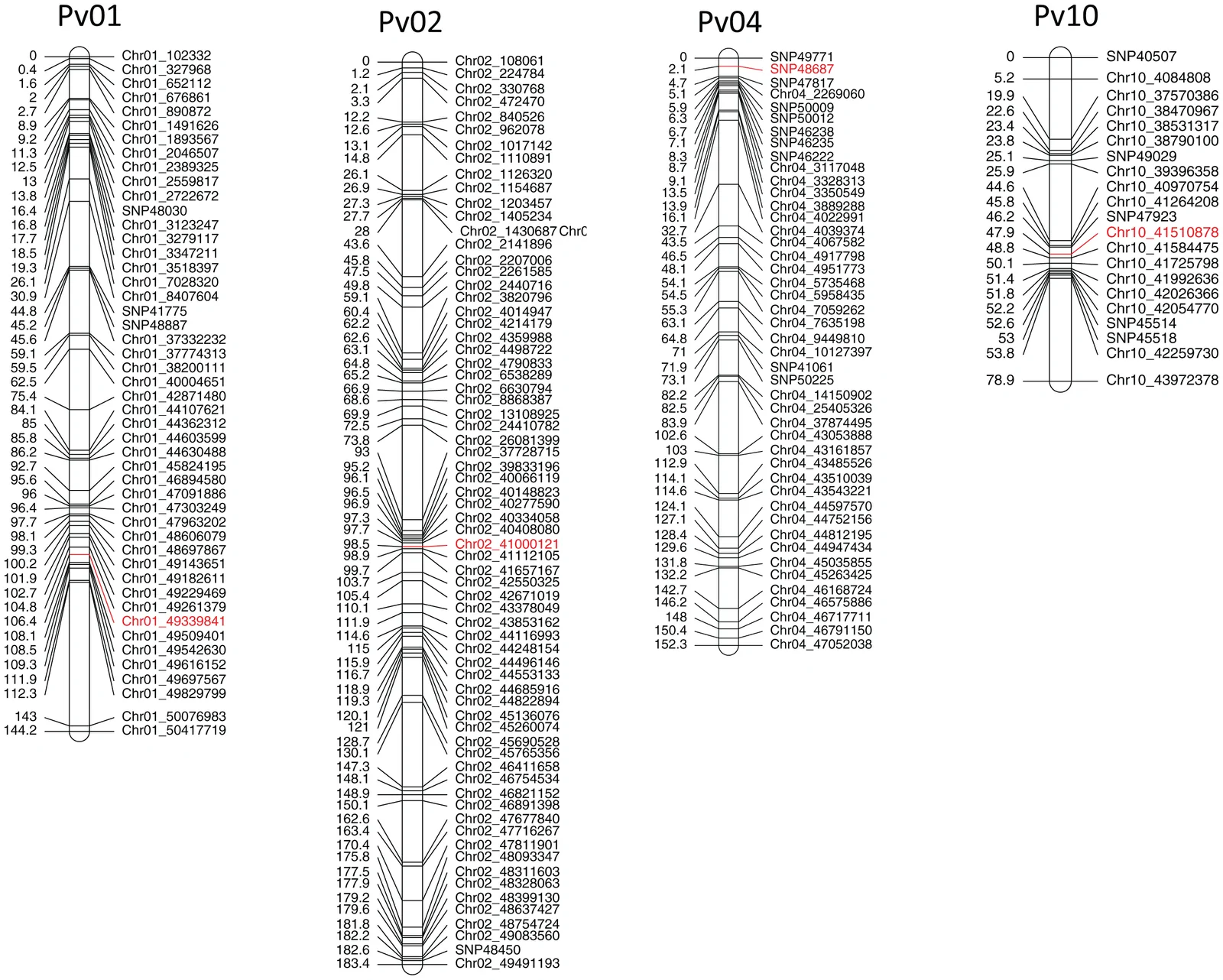
Linkage maps for four chromosomes showing QTL positions (markers in red font) for resistance to races 7 (Pv10), 15 (Pv10), 17 (Pv01), 291(Pv04) and isolate A23-13 (Pv01 and Pv02) of *C. lindemuthianum*.

ANT1.1^LM^ was identified on chromosome Pv01 for resistance to race 17 and isolate A23-13. For race 17, the QTL peaked at 49.5 Mbp (LOD = 19.3) and explained 60.5% of the phenotypic variation, with the resistance allele contributed by Mwezi Moja. For isolate A23-13, ANT1.1^LM^ peaked at 49.6 Mbp (LOD = 19.8), explained 57.7% of the phenotypic variation, and the resistance allele was also contributed by Mwezi Moja. Five genes encoding serine/threonine kinase proteins were identified as positional candidates near the ANT1.1^LM^ peak (**Supplementary Table 2**).

The second QTL, ANT2.1^LM^, was detected on Pv02 at 41.0 Mbp. This locus had a minor effect, explaining 4.2% of the phenotypic variation. Three genes identified as candidate genes associated with the ANT2.1^LM^.

The third QTL, ANT4.1LM, was located at the beginning of Pv04 (0.3 Mbp). This QTL had a major effect, explaining 75.5% of the variation among RILs in their reaction to race 291. A total of eleven disease resistance (R genes) encoding the NB-ARC-LRR were identified as candidate genes associated with the QTL ANT4.1^LM^ and occurred in a cluster (**Supplementary Table 2**).

The fourth QTL ANT10.1^LM^ was identified on Pv10 and conferred resistance to races 7 and 15. The QTL had a major effect, explaining 18.9% and 25.9% phenotypic variation in the resistance to races 7 and 15, respectively. Two genes encoding a MYB transcription factor and NB-ARC-LRR were identified as positional candidates associated with the ANT10.1^LM^ (**Supplementary Table 3**).

## Discussion

The purple speckled (Kabulangeti) and yellow bean market classes are among the most important common bean types cultivated in Africa. One of the major constraints to the production of these market classes is ANTH, caused by *C. lindemuthianum*, a pathogen characterized by high genetic variability and the occurrence of numerous physiological races. In the present study, four isolates of *C. lindemuthianum* collected from two bean-growing regions of Zambia were characterized into races 7, 15, 17, and 291, and their genetic basis of resistance was investigated in a recombinant inbred line (RIL) population derived from a cross between the purple speckled variety Mwezi Moja and the yellow landrace Lusaka.

All four races identified were mixed races, showing virulence on both Andean and Middle American differential cultivars. The presence of mixed races is not surprising given that both Andean and Middle American bean genotypes are cultivated in the regions where the isolates were collected, although Andean types predominate. Previous studies investigating the genetic diversity of *C. lindemuthianum* in Zambia have also reported substantial pathogen variability (Zulu, 2005; Kachapulula, 2010; Mungalu et al., 2020; Nalupya et al., 2021; Kuwabo et al., 2023; Sansala et al., 2024; Sinkala et al., 2024; Mulube et al., 2025; Kachapulula et al., 2025; Mulube et al., 2026b). Of the four races characterized in this study, races 7, 15 and 17 were previously been reported in Zambia (Kachapulula, 2010; Sansala et al., 2024; Mulube et al., 2026b), but race 291 is being reported for the first time in Zambia. The identification of four races from only two geographic regions is therefore consistent with earlier reports and highlights the considerable diversity of the pathogen within the Zambian bean production system. Given the race-specific nature of ANTH resistance, this diversity underscores the need for continued surveillance of pathogen populations and genetic studies aimed at identifying resistance loci that can confer durable and broad-spectrum resistance.

The phenotypic distribution of disease severity indicated that resistance to race 291 and isolate A23–13 followed a bimodal distribution pattern, suggesting the presence of qualitative resistance likely controlled by one or a few major-effect genes. Broad-sense heritability estimates for resistance to the four races were high (94.5–96.9%), indicating that genetic factors accounted for a large proportion of the observed ANTH resistance to the four races and one isolate. High heritability suggests that the resistance observed in this population can be effectively exploited in breeding programs aimed at developing ANTH-resistant bean varieties in the yellow and purple speckled common bean market classes.

Despite the broad virulence of the races evaluated, RIL LM145 remained highly resistant to all four races and A23-13, with disease severity scores below 2. LM145 has a white seed coat, whereas the parental market classes are manteca yellow and purple speckled. Its principal breeding value is therefore as a resistance donor rather than as a direct replacement for these market classes. LM145 can be crossed to yellow, purple-speckled, or other market-class parents, followed by selection in sufficiently large segregating populations to recover the desired seed-coat phenotype together with the resistance alleles. Marker-assisted selection for validated resistance loci could further facilitate recovery of resistant progeny while maintaining market-class requirements. The superior resistance of LM145 relative to both parents is consistent with transgressive segregation and makes it a useful genetic resource for broadening anthracnose resistance in common bean breeding.

The major QTL ANT1.1^LM^, which confers resistance to race 17 and isolate A23-13, overlaps with the Andean resistance locus *Co-1*. The *Co-1* locus is well known for providing strong resistance to several Andean races and, to a lesser extent, some Middle American races of *C. lindemuthianum*. Interestingly, ANT1.1^LM^ also co-localizes with the *Phg-1* locus, a major resistance locus for angular leaf spot, another economically important disease of common bean in Africa and Latin America (Gonçalves-Vidigal et al., 2011). Previous studies conducted in Zambia have reported that the *Co-1* locus confers resistance to several races of *C. lindemuthianum*, including races 6, 17, 49, 81, 385, 1050, 1105, 1141, 1153 and 1277 (Mulube et al., 2025: Kuwabo et al., 2023). The co-localization of ANTH and angular leaf spot resistance loci further highlights the importance of this genomic region for disease resistance in common bean.

The minor-effect QTL ANT2.1^LM^ was detected for resistance to isolate A23–13 and explained only 4.2% of the phenotypic variation. Importantly, its interval was very broad (2.3-47.7 Mbp), indicating substantial uncertainty in its genomic position and limited mapping resolution. ANT2.1^LM^ should therefore be regarded as a tentative minor-effect locus that requires validation in additional populations or experiments before use in marker-assisted breeding. Because of this broad interval, candidate-gene interpretation for ANT2.1^LM^ was not considered sufficiently reliable.

Another major QTL identified in this study, ANT4.1^LM^, conferred resistance to race 291 and explained a large proportion of the phenotypic variance (*R²* = 75%). This high effect is consistent with the bimodal distribution observed for disease severity, which strongly suggests the action of a single major gene. ANT4.1^LM^ overlaps with the Co-3 locus and genomic regions associated with resistance to halo blight, rust (*Ur-5, Ur-14*, and *Ur-Dorado*), and angular leaf spot (*Phg-3*) (Valentini et al., 2017; Nay et al., 2019). This genomic region therefore represents an important hotspot for multiple disease resistance in common bean. Several resistance gene analogs were identified within the ANT4.1^LM^ region, occurring in clusters that are characteristic of plant disease resistance loci. It is possible that different genes within this cluster confer resistance to different pathogen races or diseases. In Zambia, the *Co-3* locus has previously been reported to provide resistance to a wide range of races affecting both Andean and Middle American beans, including races 6, 19, 49, 51, 55, 95, 167, 183, 263, 499, 530, 566 and 1331 (Mungalu et al., 2020; Kuwabo et al., 2023; Mulube et al., 2025; Kachapulula et al., 2025; Mulube et al., 2026b). These findings further highlight the potential importance of ANT4.1^LM^ in providing resistance to diverse races of *C. lindemuthianum* as well as other major diseases affecting common bean. A total of eight disease resistance genes with the NB-ARC-LRR domains were identified as positional candidate genes for ANT1.1^LM^ and ANT4.1^LM^. These R genes occurred in clusters, a pattern that possibly could be the reason that both loci provided resistance to multiple races. It is possible that individual R genes in the clusters provide resistance to specific races. The role of R genes in disease resistance particularly vertical resistance (near-total resistance) is well documented.

A major QTL was also identified toward the distal region of chromosome Pv10. This QTL occurs in a different genomic position from other previously reported ANTH resistance loci located near the beginning of Pv10 (Lateef et al., 2024; Zuiderveen et al., 2016), but overlaps with other QTL previously reported toward the end of this chromosome (Mungalu et al., 2020; Kachapulula et al., 2025; Mulube et al., 2025).

Positional candidate genes were identified for the major QTL with comparatively narrow intervals. These included genes encoding serine/threonine kinases, NB-ARC-LRR proteins, and transcription factors such as MYB. Protein kinases play key roles in signal-transduction pathways involved in plant immune responses (Romeis, 2001; Tena et al., 2011), while MYB transcription factors are known to regulate defense-related processes (Zhang et al., 2019; Yu et al., 2023). These genes represent positional candidates based on genomic proximity and functional annotation; functional validation will be required to establish causal relationships with anthracnose resistance.

## Conclusion

Four C. lindemuthianum isolates collected from two major bean-producing regions of Zambia were characterized as races 7, 15, 17, and 291. RIL LM145 exhibited high resistance to all four races and the uncharacterized isolate A23-13, identifying it as a valuable resistance donor for common bean breeding.

Four QTL (ANT1.1^LM,^ ANT2.1^LM^, ANT4.1^LM^, and ANT10.1^LM^) were associated with anthracnose resistance in the Lusaka × Mwezi Moja RIL population. ANT1.1^LM^ and ANT4.1^LM^ co-localized with the known resistance loci Co-1 and Co-3, respectively. ANT10.1^LM^ conferred resistance to races 7 and 15, whereas ANT2.1^LM^ was a minor-effect locus with a broad interval and should be interpreted cautiously. Combining validated major resistance loci through breeding may contribute to broader resistance to diverse *C. lindemuthianum* races, although durability will require validation across pathogen populations, environments, and generations.

## Data Availability

The original contributions presented in the study are included in the article/**Supplementary Material**. Further inquiries can be directed to the corresponding author.
